# Matrix-metalloproteinase-2, -8 and -9 in serum and skin blister fluid in patients with severe sepsis

**DOI:** 10.1186/cc8938

**Published:** 2010-03-31

**Authors:** Fiia P Gäddnäs, Meeri M Sutinen, Marjo Koskela, Taina Tervahartiala, Timo Sorsa, Tuula A Salo, Jouko J Laurila, Vesa Koivukangas, Tero I Ala-Kokko, Aarne Oikarinen

**Affiliations:** 1 Department of Anesthesiology, Division of Intensive Care, Oulu University Hospital, Kajaanintie 50, Oulu, FI-90029, Finland; 2 Department of Diagnostics and Oral Medicine, Oulu University Hospital, Institute of Dentistry, University of Oulu, Aapistie 7, Oulu, FI-90014, Finland; 3 Department of Oral and Maxillofacial Diseases, Helsinki University Central Hospital, Institute of Dentistry, University of Helsinki, Mannerheimintie 172, FI-00014, Finland; 4 Department of Surgery, Oulu University Hospital, Kajaanintie 50, Oulu, FI-90029, Finland; 5 Department of Dermatology and Clinical Research Center, Oulu University Hospital and University of Oulu, Kajaanintie 50, Oulu, FI-90029, Finland

## Abstract

**Introduction:**

Matrix metalloproteinases (MMPs) have various roles in inflammatory states. They seem to be able to modulate endothelial barriers and regulate the activity of chemokines and cytokines. The timely development of the levels during severe sepsis and thereafter have not been investigated. In addition it was of interest to study alterations of MMP-levels in intact skin, as the skin is the largest barrier against external pathogens and MMPs have not been studied at organ level in human sepsis. The aim of this study was to investigate the timely development of serum and skin MMP-2, -8 and -9 levels in human severe sepsis and their association with disease severity and mortality.

**Methods:**

Forty-four patients with severe sepsis and fifteen healthy controls were included in this prospective longitudinal study. The amounts of MMP-2, -8 and -9 were analyzed from serum at days 1, 4, 6, 8, and 10, and from skin suction blister fluid at days 1 and 5 from the beginning of severe sepsis. Additionally, samples from the survivors were obtained after three and six months.

**Results:**

The levels of MMP-2 and -8 were up-regulated in severe sepsis in comparison to healthy controls in skin blister fluid and serum. Compared to the controls MMP-9 levels were lower in sepsis from the fourth day on in serum and both the first and fifth day in skin blister fluid. Active forms of MMP-2 and -9 were present only in severe sepsis. The non-survivors had higher pro- and active MMP-2 levels than the survivors in skin blister fluid samples. Furthermore, MMP-2 levels were more pronounced in blister fluid and serum samples in patients with more severe organ failures. In the survivors at 3 and 6 month follow-up the MMP levels had returned to normal.

**Conclusions:**

MMP-2 and -8 are elevated in serum and blister fluid in severe sepsis, implying that they may play a significant role in the pathogenesis of severe sepsis and organ dysfunctions. Active forms of MMP-2 and 9 were only present in patients with severe sepsis, and higher MMP-2 levels in skin blister and serum were associated with more severe organ dysfunctions.

## Introduction

Matrix metalloproteinases (MMPs) are a family of endoproteinases that have an important role in the regulation of host response, including functions in different phases of inflammation and repair. Accordingly, MMPs could play a significant role in the massive inflammatory response seen in sepsis and resultant organ dysfunctions. Few recent studies have given insight in to MMP expression in the beginning of human sepsis, but longitudinal studies of the timely development of MMP levels in patients with severe sepsis and their association to disease severity and outcome have not been conducted before. MMP levels at organ level have also not been studied in sepsis.

MMPs have been shown to regulate several phases of inflammation. For example, MMP-2 and MMP-9 have been recently suggested to participate in the cleavage of endothelial tight junction components and thus increase vascular permeability and the passage of inflammatory cells and mediators to the site of inflammation [1]. Furthermore, MMP-8 and MMP-9 can activate and MMP-2 can inactivate chemokines and thus promote recruitment and extravasation of neutrophils to the damaged tissue [2,3]. MMPs also modulate the activation of cytokines. MMP-2 and MMP-9 seem to be able to release transforming growth factor (TGF)-beta from an intracellular complex [4]. However, MMP-2, MMP-3 and MMP-9 are not only able to cleave IL-beta 1 precursor to the active form but also to attenuate the signal by degrading the active form [5,6]. MMP-8 has also been suggested to have anti-inflammatory roles in experimental mice studies [7,8]. To date there are few studies reporting the role of MMPs in the beginning of severe sepsis in humans. Nakamura and colleagues were the first to report evidence of elevated MMP-9 levels with association to mortality in sepsis [9]. Hoffmann and colleagues, demonstrated elevated plasma levels of MMP-9 and tissue inhibitors of matrix metalloproteinases (TIMP)-2, and TIMP-1 on the first day of severe sepsis and significantly higher TIMP-1 levels in non-surviving patients [10]. Recently Lorente and colleagues reported elevated MMP-10 and TIMP-1 levels in the beginning of severe sepsis [11]. Furthermore, in secondary peritonitis and consequent septic shock, the MMP-8 levels in peritoneal fluid were shown to be increased in the beginning of the disease compared with serum levels [12].

We measured the MMP-2, MMP-8 and MMP-9 levels during human severe sepsis and after recovery in serum and locally in skin using the suction blister method [13]. Skin is one of the organs affected by sepsis and is readily available for examination by relatively non-invasive methods. Its appropriate function is also of interest, because skin is the largest barrier maintaining internal homeostasis. Our hypothesis was that levels of MMPs are increased in severe sepsis both at systemic and local levels, and that the levels are associated with the severity of organ dysfunctions and outcome of the patients.

## Materials and methods

### Patients

This is a substudy of a larger study on connective tissue metabolism and wound healing in sepsis. The study group consisted of 44 patients with severe sepsis, who were prospectively followed for 10 days from the diagnosis of severe sepsis. The study was conducted in a 12-bed mixed-type adult ICU of Oulu University Hospital, Finland - an academic tertiary-level referral hospital. All patients admitted from May 2005 to December 2006 were screened. The inclusion criterion was severe sepsis with or without septic shock. These were defined according to the American College of Chest Physicians/Society of Critical Care Medicine criteria [14]. Exclusion criteria included age under 18 years, bleeding disorder, immunosuppressant therapy, surgery not related to sepsis, surgery during the preceding six months, malignancy, chronic hepatic failure, chronic renal failure and steroid therapy not related to sepsis. The patients entered the study within 48 hours after the first organ dysfunction criterion of severe sepsis was met. The patients were treated according to normal ICU protocol and severe sepsis guidelines, including steroid supplementation in septic shock. The study protocol was approved by the hospital's ethics committee and all the patients or their next of kin gave written consent for the study. Fifteen healthy adults were used as controls.

### Clinical data

The information collected from all the study patients included age, sex, chronic diseases, type of ICU admission (medical or surgical), reason for admission, focus of infection, severity of underlying diseases on admission as assessed by the Acute Physiology and Chronic Health Evaluation II (APACHE II), evolution of daily organ dysfunctions assessed by daily Sequential Organ Failure Assessment (SOFA) scores. Organ dysfunction was defined as an individual organ SOFA score of one to two and organ failure as a SOFA score of three to four. Multiple Organ Failure (MOF) was defined as daily SOFA scores of two or more organ systems three to four on one or more days during the study period. Additively Multiple Organ Dysfunction Syndrome (MODS) was defined as daily SOFA scores of one to two in two or more organ systems on one or more days [15]. The length of the ICU and hospital stays as well as the ICU, hospital and 30-day mortalities were recorded.

### Blood samples

The blood samples were obtained for MMP analysis on days 1, 4, 6, 8 and 10 in 10 ml vacuum glass tubes without clot activator. In addition, samples from survivors were also collected three and six months after recovering from the sepsis. Blood samples from the controls were collected once. After the centrifugation, the serum was frozen and stored at -70°C until the analysis.

### Suction blisters

Local MMP concentrations of the skin were assessed analyzing the suction blister fluid which closely resembles the skin interstitial fluid [16]. The skin suction blister method has first been described by Kiistala [13] and modified for measurement of MMPs by Oikarinen and colleagues [17]. The suction blisters were induced on abdominal skin using commercially available suction blister devices (Dermovac blistering device; Mucel Co., Nummela, Finland) on days one and five of the study. The device is 50 mm in diameter and contains five pores to which the suction is conducted. With prolonged suction five blisters 6 mm in diameter are formed. Instantly after the blisters were fully developed the blister fluid was collected with 18 G needle and syringe. In survivors, suction blisters were also induced three and six months after study entry. One set of suction blisters was made on the controls. The blister fluid was immediately frozen and stored at -70°C until analysis.

### Measurements of MMP-2 and MMP-9 by gelatin zymography

A 1 μL sample of serum and 2 μL of suction blister fluid were used to analyze MMP-2 and MMP -9 in 10% SDS-PAGE containing 1 mg/ml gelatin labeled fluorescently with 2-methoxy-2,4-diphenyl-3(2*H*)-furanone (Fluka, Ronkonkoma, NY, USA) [18]. Low-range prestained SDS-PAGE Standards (Bio-Rad, Hercules, CA, USA) were run in each gel as well as control MMP-2 and MMP -9 samples purified from fibroblast and keratinocyte mediums, respectively. Prior to electrophoresis, some suction blister fluid samples were incubated with 2 mM 4-aminophenylmercuric acetate (APMA, Sigma Chemical Company, St. Louis, MO, USA) at 37°C for one hour. The APMA treatment was stopped by adding the electrophoresis sample buffer. After electrophoresis, gelatinases were activated by incubating the gels for two to three hours at 37°C. As the gelatin used in the gels was fluorescently labeled the appearance of the gelatinolytic bands during incubation could be monitored under long wave UV light. The gels were stained with 0.5% Coomassie Brilliant Blue R-250 and the intensities of the bands were quantified using optical densitometry and Quantity one software (Bio Rad Model GS-700 Imaging Densitometer, Bio-Rad, Richmond, CA, USA). The intensity is expressed as densitometric units (dU).

### Immunofluorometric assay of MMP-8

The MMP-8 concentrations were determined by a time-resolved immunofluorometric assay (IFMA). The monoclonal MMP-8 specific antibodies 8708 and 8706 (Medix Biochemica, Kauniainen, Finland) were used as a catching antibody and a tracer antibody, respectively. The tracer antibody was labeled using europium-chelate [19]. The assay buffer contained 20 mM Tris-HCl, pH 7.5, 0.5 M NaCl, 5 mM CaCl_2_, 50 μM ZnCl_2_, 0.5% BSA, 0.05% sodium azide and 20 mg/l diethylenetriaminepentaacetic acid (DTPA). Samples were diluted in assay buffer and incubated for one hour, followed by incubation for one hour with tracer antibody. Enhancement solution was added and after five minutes fluorescence was measured using a 1234 Delfia Research Fluorometer (Wallac, Turku, Finland). The specificity of the monoclonal antibodies against MMP-8 corresponded to that of polyclonal MMP-8.

### Statistical analysis

Serum and blister fluid levels of MMP-8, MMP-9 (92 kDa and 82 kDa forms), and MMP-2 (72 kDa and 62 kDa forms) were compared between non-surviving and surviving patients as well as between MODS and MOF patients. The time points for the comparisons were on day 1 and 5 for blister fluid samples and days 1, 4, 6, 8 and 10 for serum samples. The serum and blister fluid MMP-levels of MODS and MOF patients were additively compared at three and six months after recovering sepsis. The comparisons of MMPs studied from blister fluid and serum were made also between septic patients and controls at each measuring point mentioned above. The summary measurements for continuous and ordinal variables were expressed as means with standard deviation or a median with 25^th ^to 75^th ^percentile. Chi-squared or Fisher's exact test was used for categorical data. Between group comparisons for continuous variables were performed using Student's t-test or Mann-Whitney U test. The linear mixed model was utilized for repeated measurement analyses when comparing MODS and MOF patients. In the mixed model approach sex, medical/surgical admission or the use of corticosteroids for the treatment of septic shock refractory to vasopressor therapy, were used one by one as an adjusting covariate if their impact on the model was significant. The *P *values are reported as follows: *P*_g_, indicates a significant level difference between the groups, *P*_t+g _indicates time-group interaction and *P*_t _indicates the change over time. The statistical analyses were performed using SPSS (SPSS, version 16.0, SPSS Inc, Chicago, IL, USA) and SAS (version 9.1.3, SAS Institute Inc., Cary, NC, USA) statistical software.

Two-tailed significance levels are reported. Readers should take into account that where several comparisons are made no *P *value correction coefficient method is used.

## Results

### Patients

Of the 1,361 patients admitted to the ICU during the period from May 2005 to December 2006, 238 adults met the inclusion criteria. One hundred and seventy-two patients were excluded and 44 of the remaining 66 patients or their next of kin gave written informed consent. The control group consisted of age- and sex-matched healthy volunteers with a median age of 60 years (25^th ^to 75^th ^percentile 56 to 68 years). Seven of them were females and eight were males. The patient demographics and clinical characteristics have been reported previously [20] and are summarized in Table 1. The overall median age was 63 years (25^th ^to 75^th ^percentile 53 to 71 years). The overall median APACHE II score at admission was 26 (22 to 30). Of the cases, 68% developed MOF and 86% required noradrenaline and 73% hydrocortisone therapy for septic shock. The non-survivors had significantly higher APACHE II score on admission and maximum SOFA scores (31 (25^th ^to 75^th ^percentile 26 to 37) vs. 24 (22 to 27), *P *= 0.005 and 16 (11 to 20) vs. 8 (7 to 11), *P *= 0.003, respectively). Lungs were the most common infection focus and blood culture was positive in 13 cases.

**Table 1:** Characteristics of the surviving and non-surviving study patients. Categorical variables are presented as frequencies with percents and other variables as medians with 25^th ^to 75^th ^percentiles

	All (n = 44)	Survivors (n = 33)	Non-survivors (n = 11)	*P*
Male sex	29 (57%)	20 (60%)	9 (80%)	0.31
Age, years	63 (53-71)	61 (56-66)	71 (62-74)	0.06
Body mass index, kg/m^2^	26 (24-32)	28 (23-33)	26 (24-27)	0.17
Chronic diseases				
-ischemic heart disease	9 (20%)	5 (15%)	4 (36%)	
-diabetes	10 (23%)	9 (27%)	1 (9%)	
-chronic obstructive pulmonary disease	5 (11%)	4 (12%)	1 (9%)	
-asthma	4 (9%)	4 (12%)	0	
Focus of infection				
-lungs	18 (41%)	13 (39%)	5 (45%)	
-intra-abdominal	16 (36%)	12 (36%)	4 (36%)	
-urinary	1 (2%)	1 (3%)	0	
-primary blood	3 (7%)	2 (6%)	1 (9%)	
-other	6 (14%)	5 (15%)	1 (9%)	
APACHE II score	26 (22-30)	24 (22-27)	31 (26-37)	0.005
Maximum SOFA score	9.5 (7-16)	8 (7-11)	16 (11-20)	0.003
Multiple organ failure	30 (68%)	20 (60%)	10 (90%)	<0.001
Length of stay (at the intensive care unit)	6.6 (4-12)	6 (4-8)	11 (6-14)	0.16
Surgical admission	25 (57%)	18 (55%)	7 (63%)	<0.001
Hydrocortisone therapy	32 (73%)	22 (67%)	10 (90%)	<0.001
Noradrenaline maximum rate, μg/kg/min	38 (86%) 0.42(0.19-1)	27 (82%) 0.25(0.09-0.43)	11 (100%) 0.96 (0.53-1.80)	0.13 0.005
Adrenaline	1 (2%)	1(3%)	0	0.56
Vasopressin and analogues	6 (14%)	3 (9%)	3 (27%)	0.15
Activated protein C	6 (14%)	3 (9%)	3 (27%)	0.13

APACHE, Acute Physiology and Chronic Health Evaluation II score; SOFA, Sequential Organ Failure Assessment.

### MMP-8, MMP-2 and MMP-9 in blister fluid in patients and healthy controls

**Figure 1:**
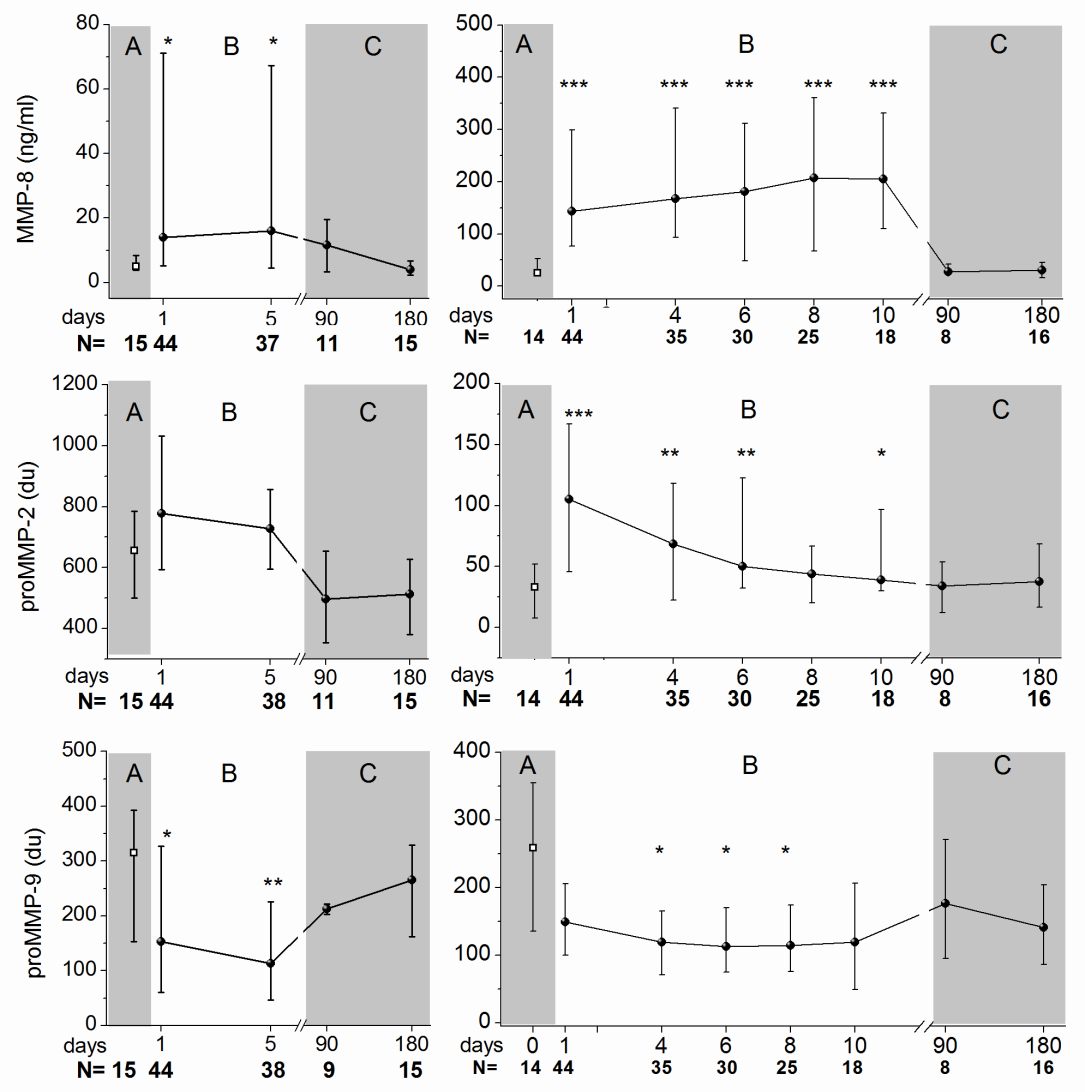
**MMP-8, proMMP-2 (62 kDa) and proMMP-9 (92 kDa) levels in patients with severe sepsis and in healthy controls**. Results from the suction blister samples are on the left and from the serum samples on the right. Panel A presents the control value, panel B the values of all the patients in severe sepsis and panel C the values of the surviving patients at three and six months after severe sepsis. The diagonal lines mark the range from 25^th ^to 75^th ^percentile. Statistically significant differences between the control values and the values of the patients at each measuring point are marked with asterisks above the values of the patients (* *P *< 0.05, ** *P *< 0.01, *** *P *< 0.001). The development of patient number (N) is expressed below the figure. MMP, matrix metalloproteinase.

**Figure 2:**
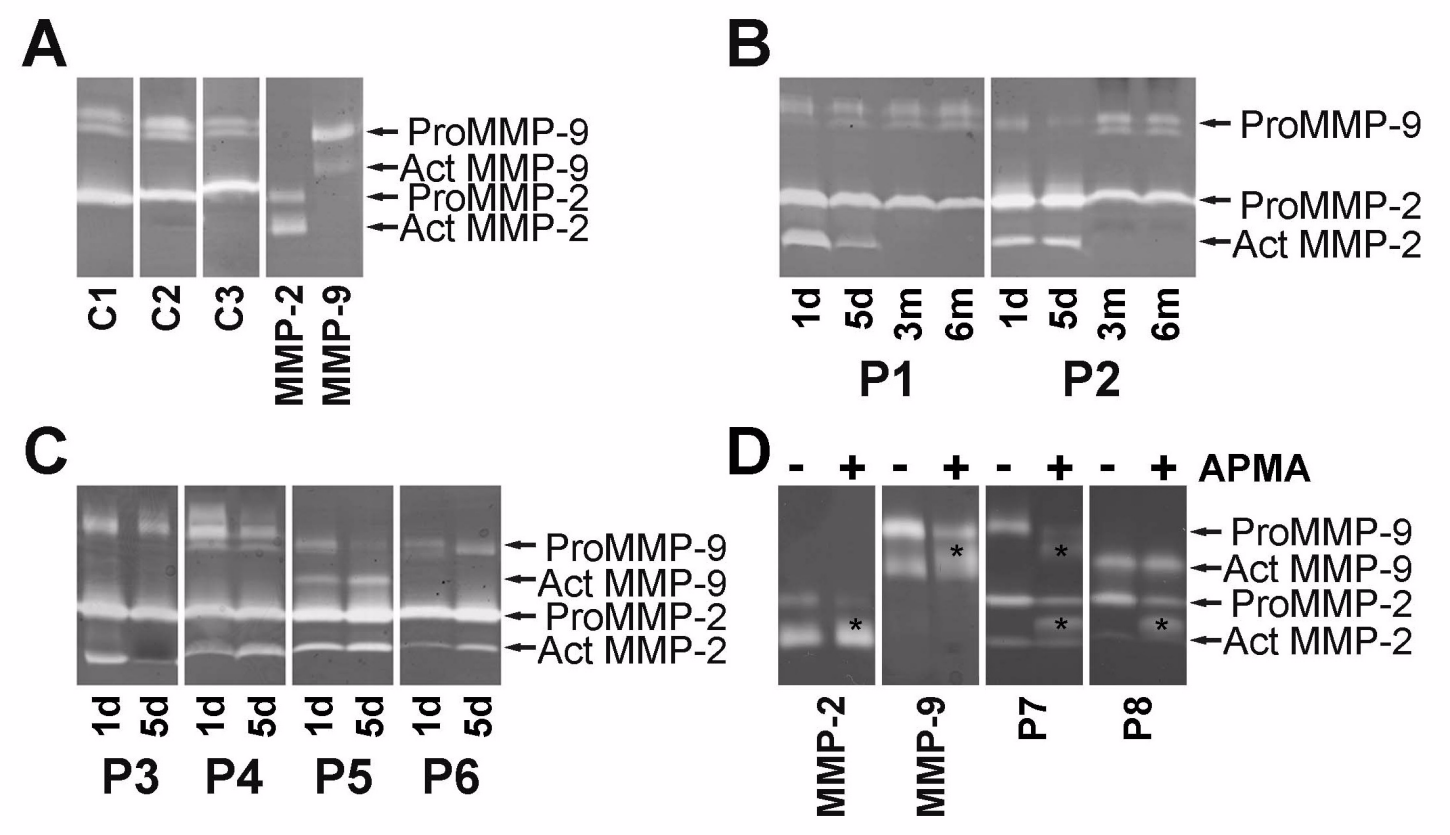
**MMP-2 and MMP -9 levels in suction blister fluids of patients with severe sepsis and healthy controls were measured by gelatin zymography**. All the gels had matrix metalloproteinase (MMP)-2 and MMP-9 samples purified from fibroblast and keratinocyte mediums, respectively. **(a) **MMP-2 and MMP-9. Pro and active forms of MMP-2 and MMP-9 are shown by arrows. As the running time for different gels varied slightly the bands are not exactly at the same level in samples analyzed in different gels. Three different healthy control samples (C1, C2, C3) are shown together with purified control MMP-2 and MMP-9. **(b) **Samples from two different surviving patients (P1, P2) are shown. For each of them one and five days and three and six month samples were run side by side in the gel. **(c) **Samples from four different non-surviving patients (P 3 to P 6) on days one and five (run side by side in the gel for each of them) are shown. **(d) **Purified control MMP-2 and MMP-9 and two different patient samples (P7, P8) incubated with (+) or without (-)4-aminophenylmercuric acetate (APMA) are shown (each sample with and without APMA was run side by side in the gel). In samples with APMA activation the bands corresponding to the proforms are weakened. Asteriks indicates the intermediate sized MMP-2 or MMP-9.

The MMP-8 levels in blister fluid samples were significantly higher in patients with severe sepsis in comparison with the controls on both days (Figure 1). The blister fluid levels of the 72 kDa proMMP-2 were slightly elevated on both study days (Figure 1). The form spliced to active conformation, the 62 kDa MMP-2, was found in all patients with severe sepsis on the first day (153.1 dU (53.2 to 373.9)) and on the fifth day (127.4 dU (47.4 to 318.2)), but not in controls (Figure 2). The 92 kDa proMMP-9 was lower on both first and fifth day in patients with severe sepsis in comparison with the controls (Figure 1). The 82 kDa MMP-9, the form spliced to active conformation, was found in blister fluid samples of five patients out of 44 on the first day and of five patients out of 38 patients on the fifth day, but not in control samples (Figure 2). Three and six months after severe sepsis no marked differences could be observed in comparison with the controls (Figure 1). Active form of MMP-2 could be detected in one of the survivors at three months, and the active form of MMP-9 in three survivors at three months and in one even at six months.

APMA is an organomercurial activator of MMPs, which converts the proMMPs into their active forms by stepwise activation. Some blister fluid samples were treated with APMA. In samples with APMA-activation the band corresponding to the proform of MMP-2 or MMP-9 weakened both in purified control MMP-2 and MMP-9 and in patient samples examined. In purified control MMP-2 and MMP-9 the band corresponding to the active form of MMP-2 or MMP-9 strengthened and a weak intermediate-sized band appeared between the pro and active forms of MMP-2 or MMP-9. In patient samples an intermediate-sized band between the pro and active forms of MMP-2 or MMP-9 appeared while the band for the active form of MMP-2 or MMP-9 was not significantly altered (Figure 2d).

### MMP-8, MMP-2 and MMP-9 in serum in patients and healthy controls

Also in the serum samples MMP-8 was found to be elevated during the ten day study period and the 72 kDa proMMP-2 was elevated until the sixth day in comparison with the controls (Figure 1). Interestingly, the 92 kDa proMMP-9 levels were lower in the serum of sepsis patients in comparison to healthy controls during the 10 days (Figure 1). The 62 kDa MMP-2 could not be detected in the serum samples in patients and controls and the 82 kDa MMP-9 could be detected only in few samples (3 on day 1; 4 on day 4; 5 on days 6, 8 and 10; and 0 at 3 and 6 months). At three and six months after the sepsis, the levels of the survivors were similar to those of the controls (Figure 1).

### Survivors in comparison with non-survivors

Blister fluid proMMP-2 levels were significantly higher in non-survivors in comparison with survivors on both first and fifth days (1132.2 dU (922.1 to 1405.1) vs. 701.99 dU (604.7 to 941.1), *P *= 0.001 and 1153.9 dU (801.9 to 1349.4) vs. 735.9 dU (627.4 to 888.6), *P *= 0.01, respectively). ProMMP-9 form in blister fluid was higher in non-survivors on the first but not the fifth day (365.4 dU (221.0 to 478.3) vs. 102.8 dU (60.8 to 273.75), *P *= 0.005 and 151.6 dU (37.5 to 231.5) vs. 127.9 dU (47.8 to 283.4), *P *= 0.84, respectively). MMP-8 levels were similar in both groups of non-survivors and survivors on both days (28.8 ng/ml (8.2 to 84.7) vs.12.8 ng/ml (5.2 to 52.8), *P *= 0.47 and 13.5 ng/ml (6.6 to 4.1) vs. 20.7 ng/ml (4.6 to 67.4), *P *= 0.84, respectively). In serum samples, there were no significant differences in the levels of MMP-8, proMMP-9 and proMMP-2 between survivors and non-survivors (data not shown).

### Patients with MODS in comparison to patients with multiple organ failure

**Figure 3:**
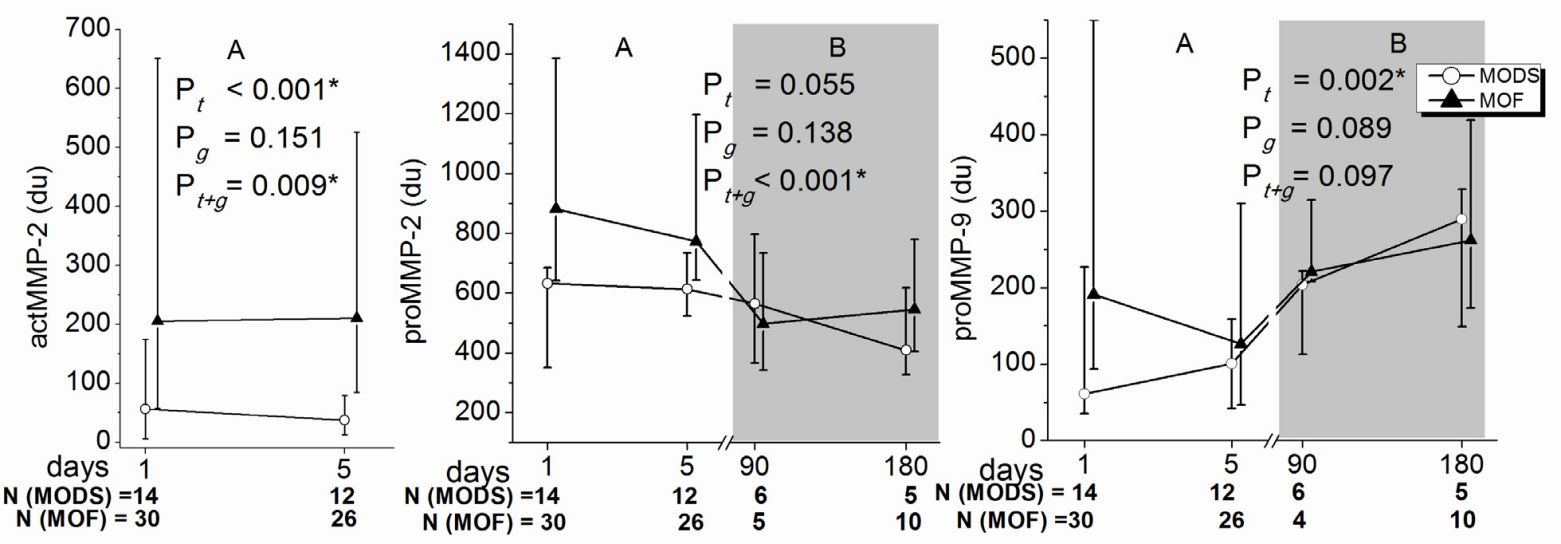
**MMP-2 (pro 72 kDa and active 62 kDa forms) and MMP-9 (pro 92 kDa form) levels in blister fluid of patients with multiple organ dysfunction syndrome (MODS) and multiple organ failure (MOF)**. Panel A presents the values of all the patients in severe sepsis and panel B the values of survivors at three and six months. *P *values from comparison of MODS and MOF patients with the linear mixed model are expressed above: *P*_g _difference between the groups, *P*_t-g _difference in time-group interaction, *P*_t _difference in change over time. MMP, matrix metalloproteinase.

Patients with MODS were compared with those having MOF with a linear mixed model. In skin blister fluid the timely development of the levels of MMP-8 did not differ between the groups during the study (data not shown). The proMMP-2 was higher on the first and fifth day in patients with MOF in comparison with MODS (935.6 dU (707.8 to 1220.8) vs. 659.3 dU (572.5 to 700.5), *P *= 0.002 and 790.0 dU (719.3 to 1092.85) vs. 641.44 dU (719.3 to 1092.85), *P *= 0.01, respectively). The active 62 kDa form was significantly higher in patients with MOF than in MODS on the first and fifth days (224.91 dU (57.1 to 502.6) vs. 69.3 dU (6.06 to 174.8), *P *= 0.03 and 239.2 dU (84.5 to 412.9) vs. 46.1 dU (18.02 to 79.3) *P *= 0.001, respectively). The proMMP-9 levels were higher in MOF than in MODS in the beginning of the study (225.2 dU (93.6 to 463.9) vs. 91.5 dU (57.7 to 227.0), *P *= 0.05; Figure 3).

**Figure 4:**
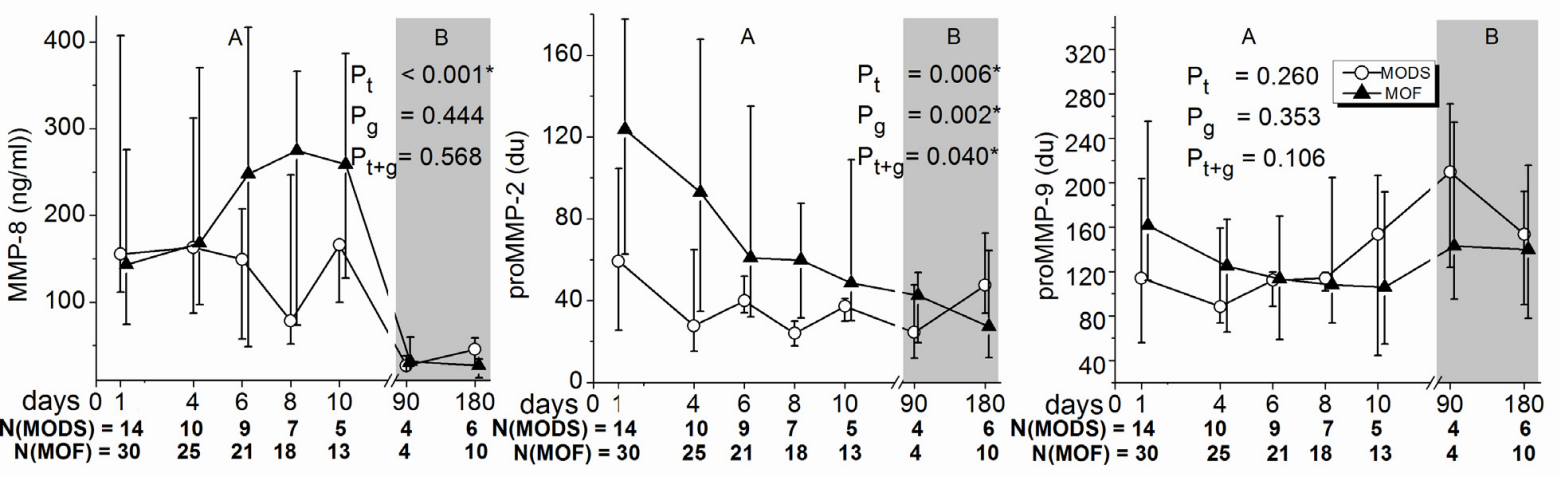
**pro MMP-2, MMP-8 and pro MMP-9 levels in serum of patients with multiple organ dysfunction syndrome (MODS) and multiple organ failure (MOF)**. Panel A presents the values of all the patients in severe sepsis and panel B the values of survivors at three and six months. *P *values from comparison of MODS and MOF patients with the linear mixed model are expressed above: *P*_g _difference between the groups, *P*_t-g _difference in time-group interaction, P_t _difference in change over time. MMP, matrix metalloproteinase.

In the serum samples the MMP-8 levels were slightly elevated from day 6 to 10 in patients with MOF compared with MODS, thus the timely development differed in these groups. The proMMP-2 values in the MOF group were higher especially at the beginning of the study. The levels and timely development of proMMP-9 did not significantly differ between patients with MOF and MODS (Figure 4).

### Correlations with organ dysfunction parameters

No correlations between APACHE II score on admission and MMP-2, MMP-8 and MMP-9 were found at any time point. Instead several positive correlations were found with the daily SOFA scores. Blister fluid proMMP-2 on the first day correlated positively with SOFA scores on days 1 to 8 and proMMP-2 on the fifth day with SOFA scores on days 1 to 10. Similarly active MMP-2 blister fluid levels on day one and five correlated with SOFA scores on several days (Table 2). Also the serum levels of proMMP-2 correlated with SOFA scores. Correlations with serum proMMP-2 on day one were found with SOFA scores from days one to five and for proMMP-2 on day four with SOFA scores from days one to six. (Table 3). No correlation between daily SOFA scores and MMP-8 levels of blister fluid or serum were found. On day one blister fluid or serum proMMP-9 did not correlate to SOFA at any time point, but the blister fluid level on the fifth day correlated negatively with SOFA on day two (-0.04, *P *= 0.03) and serum level of day four with SOFA on day one (-0.36, *P *= 0.03).

**Table 2:** Correlations between blister fluid pro-MMP2 and active MMP-2 and daily SOFA scores

		*SOFA day 1*	*SOFA day 2*	*SOFA day3*	*SOFA day 4*	*SOFA day 5*	*SOFA day 6*	*SOFA day 7*	*SOFA day 8*	*SOFA day 9*	*SOFA day10*
**proMMP-2 day 1 (dU)**	**rho**	0.570**	0.504**	0.569**	0.501**	0.522**	0.536**	0.612**	0.580**	0.392	0.216
	***p***	0.000	0.000	0.000	0.002	0.003	0.008	0.002	0.005	0.133	0.421
	**N**	44	44	38	35	31	23	23	22	16	16

**proMMP-2 day 5 (dU)**	**rho**	0.394*	0.486**	0.525**	0.495**	0.576**	0.577**	0.596**	0.676**	0.633*	0.545*
	***p***	0.014	0.002	0.001	0.004	0.001	0.006	0.004	0.001	0.015	0.044
	**N**	38	38	34	32	29	21	21	20	14	14

**actMMP-2 day 1 (dU)**	**rho**	0.576**	0.493**	0.401*	0.238	0.232	0.436*	0.439*	0.437*	0.153	0.005
	***p***	0.000	0.001	0.013	0.168	0.210	0.038	0.036	0.042	0.571	0.986
	**N**	44	44	38	35	31	23	23	22	16	16

**actMMP-2 day 5 (dU)**	**rho**	0.565**	0.540**	0.551**	0.364*	0.479**	0.537*	0.570**	0.488*	0.525	0.438
	***p***	0.000	0.000	0.001	0.040	0.009	0.012	0.007	0.029	0.054	0.118
	**N**	38	38	34	32	29	21	21	20	14	14

SOFA, Sequential Organ Failure Assesment score; MMP, matrix metalloproteinase; dU, densitometric units; rho, Spearman's rank correlation coefficient. * *P *< 0.5, **P < 0.01.

**Table 3:** Correlations between serum proMMP-2 and daily SOFA-scores

		*SOFA day 1*	*SOFA day 2*	*SOFA day3*	*SOFA day 4*	*SOFA day 5*	*SOFA day 6*	*SOFA day 7*	*SOFA day 8*	*SOFA day 9*	*SOFA day10*
**proMMP-2 day 1 (dU)**	**rho**	0.0.480**	0.0.519**	0.0.547**	0.0.490**	0.0.408*	0.0.402	0.0.342	0.0.349	0.0.207	0.0.022
	***p***	0.0.001	0.0.000	0.0.000	0.0.003	0.0.023	0.0.057	0.0.110	0.0.112	0.0.441	0.0.934
	**N**	44	44	38	35	31	23	23	22	16	16

**proMMP-2 day 4 (dU)**	**rho**	0.0.423*	0.0.468**	0.0.512**	0.0.540**	0.0.431*	0.0.480*	0.0.352	0.0.350	0.0.451	0.0.471
	***p***	0.0.011	0.0.005	0.0.002	0.0.003	0.0.022	0.0.020	0.0.100	0.0.110	0.0.079	0.0.065
	**N**	35	35	33	29	28	23	23	22	16	16

**proMMP-2 day 6 (dU)**	**rho**	0.0.343	0.0.400*	0.0.350	0.0.394	0.0.253	0.0.247	0.0.084	0.0.059	0.0.354	0.0.293
	***p***	0.0.063	0.0.028	0.0.068	0.0.051	0.0.223	0.0.308	0.0.723	0.0.805	0.0.196	0.0.290
	**N**	30	30	28	25	25	19	20	20	15	15

**proMMP-2 day 8 (dU)**	**rho**	0.0.563**	0.0.477*	0.0.517*	0.0.567**	0.0.441*	0.0.421	0.0.374	0.0.341	0.0.386	0.0.512
	***P***	0.0.003	0.0.016	0.0.012	0.0.007	0.0.040	0.0.092	0.0.126	0.0.166	0.0.155	0.0.051
	**N**	25	25	23	21	22	17	18	18	15	15

**proMMP-2 day 10 (dU)**	**rho**	0.0.472*	0.0.435	0.0.422	0.0.440	0.0.259	0.0.273	0.0.170	0.0.215	0.0.389	0.0.650*
	***P***	0.0.048	0.0.071	0.0.092	0.0.088	0.0.316	0.0.366	0.0.560	0.0.460	0.0.238	0.0.030
	**N**	18	18	17	16	17	13	14	14	11	11

SOFA, Sequential Organ Failure Assesment score; MMP. matrix metalloproteinase; dU, densitometric units; rho, Spearman's rank correlation coefficient. *P < 0.50. *P < 0.01.

## Discussion

This is the first longitudinal study reporting the levels of MMP-2, MMP-8 and MMP-9 in the patients with severe sepsis. The main findings were the levels of MMP-2 and MMP-8 were up-regulated in severe sepsis both in skin blister fluid and in the serum, MMP-2 levels were higher in skin blister fluid as well as in serum in more severe organ failures, and at three and six months the MMP levels had returned to normal.

Similar to our results, increased MMP-8 levels have also been observed in a study with peritonitis patients, the majority of who had septic shock [12]. MMP-8, also called the neutrophil collagenase, is predominantly released from neutrophilic granules upon infectious stimuli. However, in sepsis patients neutrophil infiltration to experimental skin blisters has shown to be attenuated by inflammatory mediators that down-regulate chemotactic receptors on neutrophils [21]. Hence, the increased MMP-8 levels compared with controls seen in blister fluid possibly originate from circulating and marginated neutrophils, and translocates to the blister, or arise from other known cellular sources [22]. Our studies did not reveal the source, but demonstrate, that in severe sepsis MMP-8 is up-regulated even in healthy looking skin. Additively MMP-8 is not associated with organ failure parameters thus supporting the suggestion that MMP-8 has both pro- and anti-inflammatory roles.

Surprisingly, in our data the 92 kDa proMMP-9 levels were suppressed in serum from the fourth day on and in the suction blister fluid from the first day. Even when active and pro forms were calculated together the levels were suppressed in sepsis in comparison with the control samples (data not shown). Previously elevated MMP-9 levels have been reported within 24 hours from severe sepsis diagnosis [9-11]. We collected the first samples within 48 hours from the beginning of the disease. The MMP-9 levels have been shown to peak early in lipopolysaccharide and *Escherichia coli*-induced inflammatory response and return to normal within 24 hours [23,24]. In the largest of previous patient samples MMP-9 was not significantly higher in sepsis patients and a negative correlation was found to organ failure parameters [11]. This is in accordance with our results from the first study day. Our results on lower levels of MMP-9 from study day four are on another hand a novel finding. Forms spliced to active MMP-9 could be found in a few patient samples but not in controls, implying that MMP-9 had been processed, whereas from day four onwards, the proMMP-9 levels dropped in a regulative fashion. Taken together, it seems that the MMP-9 levels are elevated at the very early phase of severe sepsis, but the levels drop later on.

We found low MMP-9 levels also in skin blister fluid samples of patients with severe sepsis in comparison with the controls. This is in accordance with the growing body of evidence suggesting that neutrophil migration to tissues is impaired in sepsis [25]. The interesting finding that MMP-9 levels were higher in non-survivor sample in the blister fluid at only the first day might be due to sepsis-induced damage on the structures of healthy looking skin, observed clinically as edema and even as spontaneous blistering in most severe forms of sepsis. This hypothesis is supported by the findings that elevated MMP-9 levels have been shown in spontaneous blistering diseases and that MMP-9 during tissue healing seems to enable migration of epithelial cells by degrading collagen IV, an important component of dermoepidermal junctions [17]. In blister fluid samples of healthy looking skin the proMMP-2 form was elevated and the active form was found constantly in sepsis, but not in control samples. This is surprising in the light of previous evidence that shows that MMP-2 expression is absent in healthy skin except some sweat glands, hair follicles and macrophages [26]. The factors that have been shown to induce MMP-2 expression in human skin include skin injury [26], TNF-alpha, and TGF-beta [27]. In addition, endothelial damage and reactive oxygen species present in sepsis can trigger the activation of MMP-2. Elevated concentrations of MMP-2 are associated with septic organ damage in skin, heart and lung [28-30]. However MMP-2 seems to have both beneficial and detrimental roles in inflammation. Based on our data, the levels of MMP-2 in blister fluid samples were higher in non-survivors and we have previously shown that re-epithelization of blister wounds is delayed in non-surviving severe sepsis patients [28].

Some medications used in sepsis, including vasopressor agents, hydrocortisone and activated protein C (APC), have been shown to affect MMP expression [29,31-33]. The elimination of these clinically central therapies from a study setting with patients with severe sepsis would be impossible, and thus their role must be acknowledged when evaluating the results. In this study 86% of patients received noradrenaline, 73% hydrocortisone and 14% APC. In an ovine model of septic cardiac failure, MMP-2 levels were shown to be even higher in noradrenaline-masked hypovolemia added to endotoxemia than in endotoxemia alone [29]. APC reduced the MMP-9 levels in fibroblasts and monocytes of arthritis patients, but up-regulated and activated MMP-2 [32]. In human keratinocytes APC enhanced the expression and activation of MMP-2, but had no effect on MMP-9 [31].

This study is limited by the fact that the precise phase of inflammation was not determined on the molecular level, but from the beginning of the organ failure. This would be beneficial in the future studies, because the timing of up- and down-regulation of different inflammatory mediators will help to create a more coherent understanding on the events of septic host response. Secondly, we used healthy controls instead of critically ill patients. Systemic inflammatory response can be activated also from other reasons than infectious insult and is very common in ICU patients, especially in surgical ICUs [34]. Thus we considered it more reasonable to use healthy controls. Thirdly the number of patients was too small for a reliable statement about MMPs as prognostic markers in patients with sepsis.

## Conclusions

In severe sepsis, from intact skin suction blister and serum samples, MMP-2 and MMP-8 levels are elevated, whereas MMP-9 is suppressed. Active forms of MMP-2 and MMP-9 are only found in some patients with severe sepsis, but not in controls. The non-survivors had higher pro and active MMP-2 levels in the skin blister fluid than the survivors, and MMP-2 levels both in serum and skin blister fluid were more pronounced in patients with more severe organ failures.

## Key messages

• Levels of MMP-2 and MMP-8 were up-regulated in severe sepsis in comparison with healthy controls, both in skin blister fluid and in the serum, whereas MMP-9 levels were lower in serum in sepsis from the fourth day onwards.

• Non-surviving patients had higher MMP-2 levels in skin blister fluid during sepsis than survivors. Furthermore, MMP-2 levels were more pronounced in skin blister fluid as well as in serum in more severe organ failures.

• MMP-2 levels in serum and blister fluid correlated with daily SOFA scores.

• At the follow-up samples from surviving patients at three and six months the levels of MMP-2, MMP-8 and MMP-9 were near to normal.

## Abbreviations

APACHE II: Acute physiology and Chronic health evaluation II; APC: activated protein C; BSA: bovine serum albumin; DTPA: diethylenetriaminepentaacetic acid; IFMA: immunofluorometric assay; IL: interleukin; MMP: matrix metalloproteinase; MODS: multiple organ dysfunction syndrome; MOF: multiple organ failure; SOFA: sequential organ failure assessment; TGF beta: transforming growth factor beta; TNF alpha: tumor necrosis factor alpha.

## Competing interests

The authors declare that they have no competing interests.

## Authors' contributions

FG, MM, TS, VK, JJ, TA and AO participated in the study design. FG and MK collected the data. MM, TT and TS provided the laboratory analyses. FG performed statistical analysis and drafted the manuscript with TA and AO. AO provided the equipment for the suction blister method. All authors helped to form the manuscript and read and approved the final manuscript.
